# A case report of Bow Hunter’s syndrome with intravascular ultrasound showing changing significant severe stenosis of the left vertebral artery associated with turning left

**DOI:** 10.1093/ehjcr/ytad639

**Published:** 2023-12-22

**Authors:** Sasaki Takafumi, Rintaro Hojo, Takaaki Tsuchiyama, Seiji Fukamizu

**Affiliations:** Department of Cardiology, Tokyo Metropolitan Hiroo Hospital, 2-34-10, Ebisu, Shibuya, Tokyo 150-0013, Japan; Department of Cardiology, Tokyo Metropolitan Hiroo Hospital, 2-34-10, Ebisu, Shibuya, Tokyo 150-0013, Japan; Department of Cardiology, Tokyo Metropolitan Hiroo Hospital, 2-34-10, Ebisu, Shibuya, Tokyo 150-0013, Japan; Department of Cardiology, Tokyo Metropolitan Hiroo Hospital, 2-34-10, Ebisu, Shibuya, Tokyo 150-0013, Japan

**Keywords:** Case report, Bow Hunter’s syndrome, Intravascular ultrasound

## Abstract

**Background:**

Bow Hunter’s syndrome is vertebral basilar artery insufficiency caused by mechanical occlusion of the vertebral artery during head rotation. This is often due to the formation of osteophytes, herniated discs, cervical spondylosis, or tumours. However, whether the contralateral vessel is organically stenotic is not well known.

**Case summary:**

A 79-year-old man was referred to our department for a close examination of syncope because the transient loss of consciousness occurring when he was made to turn his head to the left was reproducibly induced and recovered when his face was returned to the normal position. The carotid massage did not induce significant bradycardia or hypotension bilaterally, and Holter electrocardiography, echocardiography, head-up tilt test, coronary angiography, and an acetylcholine stress test showed no obvious abnormalities. A 3D CT angiography was performed to investigate the possibility of vertebrobasilar artery insufficiency, as C3/4 cervical spondylosis, and the left vertebral artery was compressed by the C4 superior process osteophyte, indicating hypoplasia of the contralateral vertebral artery. Vertebral artery angiography and intravascular ultrasound (IVUS) showed moderate stenosis of the left vertebral artery, and IVUS showed a half-circumferential calcified lesion. Compared to the midline position, the stenosis worsened at the site of compression and drainage when the patient turned left downward, and a diagnosis of Bow Hunter’s syndrome was made.

**Discussion:**

Bow Hunter’s syndrome is characterized by vertebrobasilar insufficiency. Intravascular ultrasound clearly showed that the lesion was not only stenotic due to compression but also had plaque growth due to continuous mechanical stimulation.

**Learning points**

- Compression by C4 osteophytes and hypoplasia of the right vertebral artery caused severe stenosis of the left vertebral artery, leading to Bow Hunter’s syndrome.
- Intravascular ultrasound revealed plaque proliferation, possibly due to continuous mechanical stimulation.

## Introduction

Bow Hunter’s syndrome—rotational vertebral artery syndrome—is caused by mechanical occlusion of the vertebral artery during head rotation. This is often due to the formation of osteophytes, herniated discs, cervical spondylosis, or tumours.^1^ In most cases, one side has a hypoplastic vertebral artery,^2^ and the contralateral side has pressure-induced stenosis, resulting in transient brain ischaemia. However, whether the contralateral vessel is organically stenotic is not well known.

## Summary figure

**Table:** 

Time	Event
−10 years	C3/4 cervical myelopathy.
−1 month	Syncope for the first time and then repeatedly blacked out.
0 month	Presentation at our hospital.
	The transient loss of consciousness occurring when he was made to turn his head to the left was reproducibly induced and recovered when his face was returned to the normal position.
1 month	Diagnosis of Bow Hunter’s syndrome by angiography and intravascular ultrasound.
	The patient declined invasive treatment.
	Conservative treatment (cervical collar and antiplatelet therapy) started.
1 year	No recurrence of syncope.

## Case presentation

**Figure 1:**
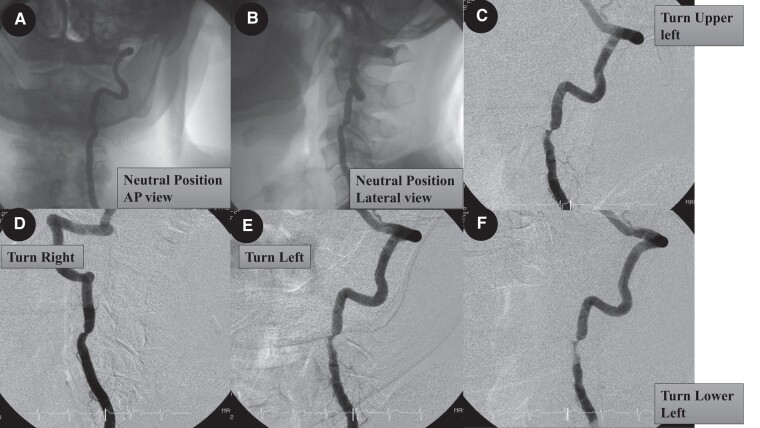
Dynamic angiography of the left vertebral artery. (*A* and *B*) The patient’s blood flow is in the neutral position. Frontal and lateral views. (C and *D*) Digital subtraction angiography (DSA) with mechanical compression on vessels on the left rather than the right side. (*E*) DSA of the vertebral artery when turned upward to the left side. (*F*) DSA of the vertebral artery with the vertebral artery facing the left downward side.

**Figure 2:**
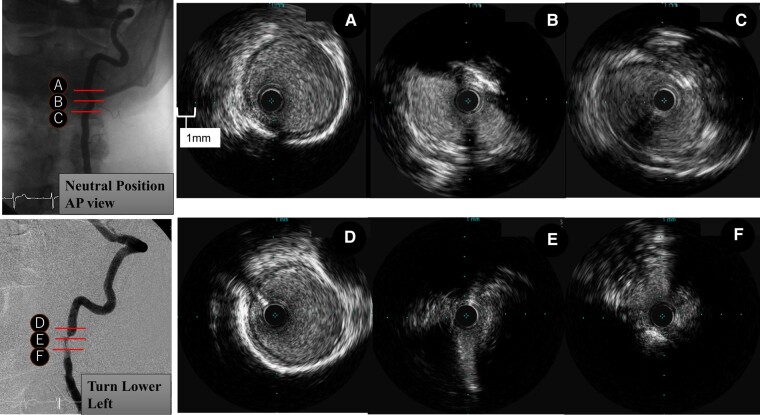
Intravascular ultrasound (IVUS) of the vertebral artery. (*A* and *B*) IVUS of the left vertebral artery in the normal cephalic position [*A*: normal site, *B*: the most stenotic site (calcified lesion at ∼90°), *C*: a partially attached plaque]. (*D–F*) IVUS with left inferior rotation (*D*: normal site, *E*: a highly stenotic lesion with the calcified lesion, (*F*): plaque adhesion with worsened stenosis).

A 79-year-old man visited his home doctor with a chief complaint of transient loss of consciousness and seizures. He was referred to our department for close examination because he experienced a transient loss of consciousness when he turned his head to the left side and recovered when his face returned to its normal position. He presented in a clinically stable condition with a blood pressure of 120/76 mmHg, a pulse of 71/min, and an oxygen saturation of 99%. The patient had a history of C3/4 cervical myelopathy, post-operative lumbar spinal stenosis, and early gastric cancer. There was no evidence of coronary artery disease, coronary angina pectoris, valvular disease, or arrhythmia. He could not undergo a head-up tilt test owing to back pain. Neurally mediated syncope, especially carotid sinus syndrome, was ruled out due to the negative results of bilateral carotid sinus massage. Magnetic resonance imaging of the head showed no obvious high-signal areas, and cerebral perfusion scintigraphy showed mild hypoperfusion in the right frontal to parietal lobes, but no disease-specific hypoperfusion was observed. Three-dimensional CT angiography (3D-CTA) of the head showed hypoplasia of the right vertebral artery and severe stenosis of the left vertebral artery due to compression by the C4 osteophyte. There were no abnormal anatomic findings, such as moyamoya vessels. The 3D-CTA of the head was performed in the left-rotated head position, and severe stenosis before the transverse process of C3 was found due to compression of the superior process of C4 osteophyte. Vertebral artery angiography revealed 75% stenosis of the left vertebral artery, even in the anterior position (*Figures 1* and *2*). Intravascular ultrasound (IVUS) showed semi-circumferential calcification and an atherosclerotic lesion (*Figure 2*). The left vertebral artery was highly stenosed, especially when the patient faced down on the left side (*Figure 2*). The left vertebral artery was compressed by the fourth cervical vertebral osteophyte, resulting in atherosclerosis, and the left vertebral artery stenosis was aggravated by cervical rotation to the left downward side, leading to extensive cerebral ischaemia and loss of consciousness. The patient was diagnosed with Bow Hunter’s syndrome secondary to C4 osteophyte-associated compression and severe stenosis of the left vertebral artery. Conservative, orthopaedic, and endovascular treatments were suggested.^2–6^ The patient did not wish to undergo invasive treatment and was followed up with a cervical collar. The patient was continued on antiplatelet agents and statins due to carotid artery stenosis with plaque. The syncope attacks ceased after the administration of conservative treatment and the use of a cervical collar. No syncope symptoms were observed during the two-year follow-up period.

## Discussion

Bow Hunter’s syndrome is a vertebrobasilar artery insufficiency caused by mechanical occlusion of the vertebral artery during head rotation.^1^ It is often caused by the formation of osteophytes, herniated discs, cervical spondylosis, or tumours. In the present case, the vertebral artery on one side was hypoplastic and the contralateral side was stenotic due to compression, likely leading to transient brain ischaemia. However, whether the contralateral vessel is organically stenotic is unknown. In this study, IVUS revealed the stenosed lesion due to compression and plaque growth, possibly due to continuous mechanical stimulation. In the case of a stenotic lesion of a cervical vessel, an occluded fold or one that belongs to a side may indicate Bow Hunter’s syndrome. Conservative therapy was administered, and a cervical collar was introduced, which stopped the syncope attacks. Cervical collars effectively prevent stroke and maintain neurological function in approximately half of the patients.^7^ However, IVUS revealed, plaque proliferation; in cases of recurrent syncope or when syncope occurs with milder head rotation, vascular stenosis may progress, necessitating catheterization or other treatments.^2,3^

## Conclusion

This case of Bow Hunter’s syndrome was caused by severe stenosis of the left vertebral artery due to compression by C4 osteophytes and hypoplasia of the right vertebral artery. Intravascular ultrasound revealed the possibility of plaque growth due to continuous mechanical stimulation.

## Data Availability

The data included in this article will be shared upon reasonable request to the corresponding author.

## Lead author biography

Takafumi Sasaki was born in Tokyo, Japan, in 1993. He received an MD degree from St. Marianna University School of Medicine, Kanagawa, Japan, in 2018, and completed 2 years of Japanese general residency training at Tokyo Metropolitan Ohkubo Hospital. Since 2020, Dr Sasaki has been working as a cardiologist at Tokyo Metropolitan Hiroo Hospital.

**Consent:** The authors confirm that written consent for the submission and publication of the case report was obtained from the patient in line with the COPE guidelines.

**Funding:** The authors received no specific funding for this work.
